# LIF promotes tumorigenesis and metastasis of breast cancer through the AKT-mTOR pathway

**DOI:** 10.18632/oncotarget.1772

**Published:** 2014-02-11

**Authors:** Xiaoyan Li, Qifeng Yang, Haiyang Yu, Lihua Wu, Yuhan Zhao, Cen Zhang, Xuetian Yue, Zhen Liu, Hao Wu, Bruce G. Haffty, Zhaohui Feng, Wenwei Hu

**Affiliations:** ^1^ Department of Radiation Oncology, Rutgers Cancer Institute of New Jersey, Rutgers State University of New Jersey, New Brunswick, NJ, USA; ^2^ Department of Breast Surgery, Qilu Hospital, Shandong University, Ji'nan, China; ^3^ First Affiliated Hospital, Zhejiang University, Hangzhou, China; ^4^ Department of Pharmacology, Rutgers State University of New Jersey, New Brunswick, NJ, USA

**Keywords:** LIF, breast cancer, AKT, mTOR, metastasis

## Abstract

Leukemia inhibitory factor (LIF) is a multi-functional cytokine protein. The role of LIF in tumorigenesis is not well-understood. Here, we found that LIF promotes tumorigenesis and metastasis of breast cancer. LIF promotes cell proliferation and anchorage-independent growth of breast cancer cells *in vitro*, and the growth of xenograft breast tumors *in vivo*. LIF also promotes invasion and migration of breast cancer cells *in vitro* and metastasis of breast cancer *in vivo*. We found that LIF activates the AKT-mTOR signaling pathway to promote tumorigenesis and metastasis of breast cancer. Inhibiting the AKT activity can largely block the activation of the mTOR pathway by LIF, suggesting that LIF activates the mTOR pathway through AKT. Inhibiting the AKT activity as well as inhibiting the mTOR activity largely block the promoting effect of LIF on tumorigenesis and metastasis. Furthermore, overexpression of LIF is significantly associated with a poorer relapse free survival in breast cancer patients. Taken together, our data strongly suggest that LIF plays an important role in the tumorigenesis and metastasis of breast cancer, and could be an important prognostic marker for breast cancer.

## INTRODUCTION

Breast cancer is one of the most common malignant tumors and the second most common cause of cancer related deaths in the United States. Although the death rate of breast cancer has decreased with advances in prevention, surgical resection and adjuvant therapies, there are still approximately 232,340 new cases and 39,620 deaths of breast cancer in the United States in 2013 [1]. Metastasis to vital organs such as lung, brain and bone is a major cause of death from breast cancer [2]. Therefore, there is an urgent need to further understand the molecular mechanisms underlying breast cancer tumorigenesis and metastasis.

Leukemia inhibitory factor (LIF), a member of the interleukin-6 cytokine superfamily, is a multi-functional protein that exerts different functions in different tissues/cells or under different conditions through the activation of different signaling pathways. Transduction of LIF signaling occurs through LIF's binding to LIF receptor complex composed of LIF receptor (LIF-R) and glycoprotein gp130 [3, 4], which in turn activates selective signaling pathways, including JAK/STAT3, PI3K/AKT, MAPK, and/or ERK1/2 pathways [5-10]. While the overexpression of LIF has been observed in several types of cancers including breast cancer [8, 11-15], the role of LIF in cancer is not well-understood. Limited studies suggested the potential complex role of LIF in cancer depending upon the types of the cancer. LIF could inhibit the differentiation and promote proliferation in some cancers and cancer cell lines, and has been suggested to contribute to the progression of malignancies, including rhabdomyosarcoma, choriocarcinoma and melanoma [8, 16-18]. Meanwhile, LIF has also been reported to induce the differentiation of murine myeloid leukemia cells and inhibit proliferation and growth in some other cancer cell lines [19-21]. Currently, the detailed function of LIF in breast cancer remains unclear.

In this study, we investigated the potential role of LIF in breast cancer. LIF promotes cell proliferation and anchorage-independent growth in soft agar of breast cancer cells *in vitro*, and the growth of xenograft breast tumors *in vivo*. LIF also promotes invasion and migration of breast cancer cells *in vitro* and metastasis of breast cancer cells *in vivo*. Interestingly, we found that LIF activates the AKT-mTOR signaling pathway. Importantly, the activation of AKT-mTOR signaling by LIF largely mediates the promoting effect of LIF on tumorigenesis and metastasis. Furthermore, overexpression of LIF is significantly associated with a poorer relapse free survival in breast cancer patients. Together, data from this study strongly suggest that LIF plays an important role in promoting the tumorigenesis and metastasis of breast cancer.

## RESULTS

### LIF promotes metastasis of breast cancer

**Figure 1:**
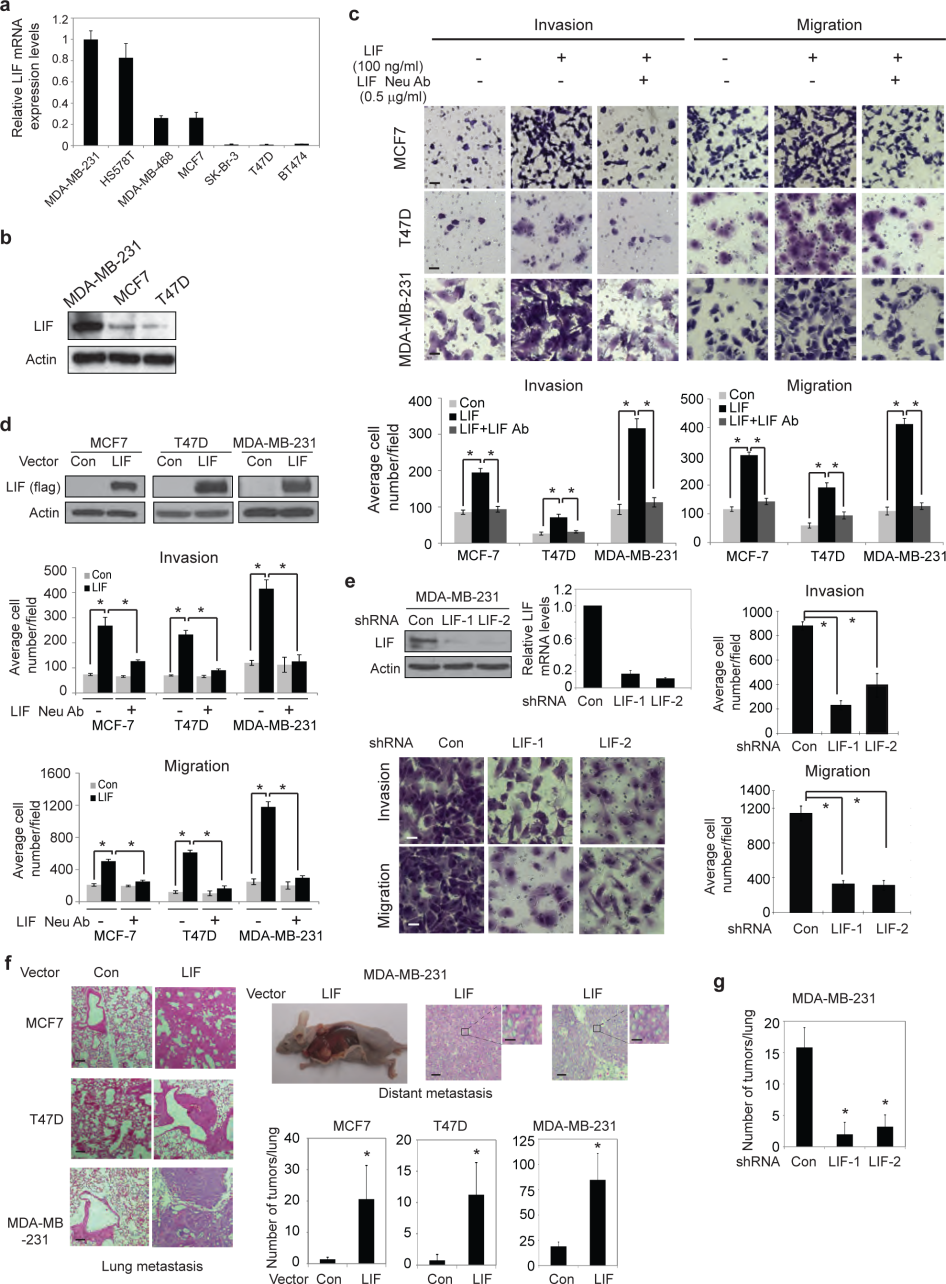
LIF promotes metastasis of breast cancer cells (a) The mRNA expression levels of LIF in a panel of human breast cancer cells were determined by Taqman real-time PCR and normalized with actin. (b) The protein levels of LIF were determined in MDA-MB-231, MCF7 and T47D cells. (c) Exogenous LIF (100 ng/ml) promoted the abilities of invasion and migration of breast cancer cells, and this effect could be blocked by LIF neutralization antibody (0.5 μg/ml) as determined by trans-well assays in chambers coated with matrigel (for invasion assays) or without matrigel (for migration assays). Upper panels: representative images; lower panels: quantifications of average number of cells/field. (d) Ectopic LIF expression promoted the invasion and migration abilities of breast cancer cells. Upper panels: Ectopic LIF expression in cells detected by Western-blot assays. (e) Knock-down of endogenous LIF reduced the invasion and migration abilities of MDA-MB-231 cells. Top left panel: The efficient knockdown of LIF mRNA and protein in cells. (f) Ectopic LIF expression promoted metastasis of breast cancer cells *in vivo*. MCF7-LIF, T47D-LIF, MDA-MB-231-LIF and their control cells (Con) were injected into nude mice *via* tail vein, and the number of metastatic tumors in lung and distant metastatic tumors was determined after 10 weeks. Representative H&E images of lung sections (left panels) and distant metastatic tumors (right top panels) are shown. (g) Knockdown of LIF reduced lung metastasis *in vivo.* MDA-MB-231-LIFshRNA and MDA-MB-231-ConshRNA cells were injected into nude mice *via* tail vein, and the number of lung metastatic tumors was determined after 12 weeks. Data are presented as mean ± SD (n=3 for a, c, d & e; n=6 for f & g). Scale bar: c & e: 50 μM; f: 50 μM for low magnitude, and 5 μM for high magnitude *: *p*<0.001.

To investigate the role of LIF in breast cancer, the mRNA levels of LIF, LIFR and gp130 were measured in a panel of human breast cancer cell lines, including MDA-MB-231, HS578T, MDA-MB-468, MCF7, SK-Br-3, T47D and BT474 cells, by employing Taqman real-time PCR assays. Whereas the majority of these cells express LIF receptors (LIFR and gp130) at relatively similar levels (Supplementary Fig. 1a&b), the expression levels of LIF varied dramatically among these cell lines, which are correlated with the metastatic abilities of these cell lines (Fig. 1a). The expression levels of LIF are much higher in MDA-MB-231 and HS578T cells that display higher metastatic abilities [22] compared to less metastatic breast cancer cells, including MCF7, MDA-MB-468, SK-Br-3, T47D and BT474 cells. The difference in LIF expression levels was confirmed at the protein level in MDA-MB-231, MCF7 and T47D cells (Fig 1b), which were chosen for the following experiments to study the role of LIF in breast tumorigenesis.

To directly investigate the effect of LIF on metastasis in breast cancer, two chamber trans-well assays were employed to determine the effects of LIF on the abilities of invasion and migration of MDA-MB-231, MCF7 and T47D cells. As shown in Fig. 1c, treating cells with exogenous LIF (100 ng/ml) dramatically promoted the abilities of invasion and migration of all these 3 breast cancer cell lines, which can be blocked by LIF neutralization antibody. To investigate whether autocrine secretion of LIF from cells has a similar promoting effect on invasion and migration of cells, MCF7, T47D and MDA-MB-231 cells with stable ectopic expression of LIF (MCF7-LIF, T47D-LIF and MDA-MB-231-LIF) were established by transduction of LIF-flag expression vectors (Fig. 1d). Compared with control cells transduced with control vectors, ectopic LIF expression greatly increased the abilities of invasion and migration of MCF7, T47D and MDA-MB-231 cells. Furthermore, this promoting effect of LIF can be blocked by LIF neutralization antibody (Fig. 1d). Knock-down of endogenous LIF by two shRNA targeting LIF in MDA-MB-231 cells, which have high endogenous LIF levels, clearly decreased the abilities of invasion and migration of cells (Fig. 1e). These results demonstrate that LIF promotes the abilities of invasion and migration of breast cancer cells in both autocrine and paracrine manners.

The effect of LIF on metastasis was further investigated *in vivo* by employing the *in vivo* lung metastasis assays. Tail vein injection of MCF7, T47D and MDA-MB-231 cells can all lead to the formation of lung metastatic tumors in mice. As shown in Fig. 1f, ectopic LIF expression in MCF7, T47D and MDA-MB-231 cells significantly increased the number of lung metastatic tumors. Furthermore, ectopic LIF expression in T47D and MDA-MB-231 cells promoted distant metastasis. Two out of six mice injected with T47D-LIF cells developed metastatic breast tumors in the neck and muscle in addition to lung tumors, and two out of six mice injected with MDA-MB-231-LIF cells developed metastatic breast tumors in mediastinum, neck, back, underarm and muscle in addition to lung tumors. In contrast, no distant metastatic tumor was observed in mice injected with T47D-Con and MDA-MB-231-Con cells within the same time period (Fig. 1f). Consistently, mice injected with MDA-MB-231 cells with stable knock-down of LIF (MDA-MB-231-LIFshRNA) formed much less metastatic lung tumors compared to mice injected with MDA-MB-231-ConshRNA cells (Fig. 1g).

### LIF promotes proliferation, anchorage-independent growth of breast cancer cells and growth of xenograft breast tumors

**Figure 2:**
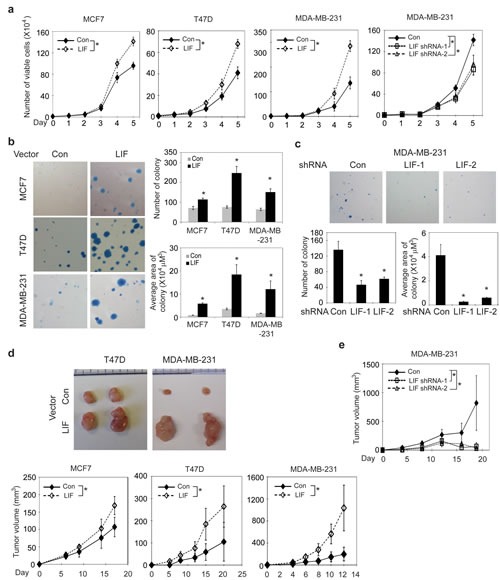
LIF promotes proliferation and anchorage-independent growth of breast cancer cells and promotes the growth of xenograft breast tumors (a) Ectopic LIF expression promoted proliferation of MCF7, T47D and MDA-MB-231 cells. Knockdown of endogenous LIF inhibited the growth of MDA-MB-231 cells. (b) Ectopic LIF expression promoted anchorage-independent growth in soft agar of breast cancer cells. Left panels: Representative images of colonies in soft agar. Right panels: average number of colonies (upper) and average area of colonies (lower). (c) Knockdown of LIF inhibited anchorage-independent growth in soft agar of MDA-MB-231 cells. (d) Ectopic LIF expression promoted the growth rate of xengoraft breast tumors. BALB/c nude mice were inoculated (s.c.) with MCF7-LIF, T47D-LIF, MDA-MB-231-LIF and their control cells (Con). Upper panels: Representative images of xenograft tumors; lower panels: growth curves of xenograft tumors. (e) Knockdown of LIF reduced the growth rate of MDA-MB-231 xenograft tumors. Data are presented as mean ± SD (n=3 for a-c; n=10 for d & e). *: *p*<0.001.

In addition to promoting metastasis, LIF also promoted proliferation of breast cancer cells. Ectopic LIF expression promoted the proliferation of MCF-7, T47D and MDA-MB-231 cells, whereas knockdown of endogenous LIF significantly inhibited the growth of MDA-MB-231 cells (Fig. 2a). Furthermore, LIF promoted the anchorage-independent cell growth in soft agar; ectopic LIF expression increased the number and size of colonies formed by MCF7, T47D and MDA-MB-231 cells (Fig. 2b), whereas knock-down of endogenous LIF inhibited the anchorage-independent growth in soft agar of MDA-MB-231 cells (Fig. 2c). Consistent with the results obtained from *in vitro* assays, ectopic LIF expression promoted the growth of xenograft tumors formed by MCF7, T47D and MDA-MB-231 cells (Fig. 2d), whereas knockdown of endogenous LIF reduced the growth of MDA-MB-231 xenograft tumors (Fig. 2e). Together, these results demonstrate that LIF promotes proliferation, anchorage-independent growth of breast cancer cells and the growth of xenograft breast tumors.

### LIF activates the mTOR pathway in breast cancer cells which contributes to the promoting effect of LIF on metastasis

**Figure 3:**
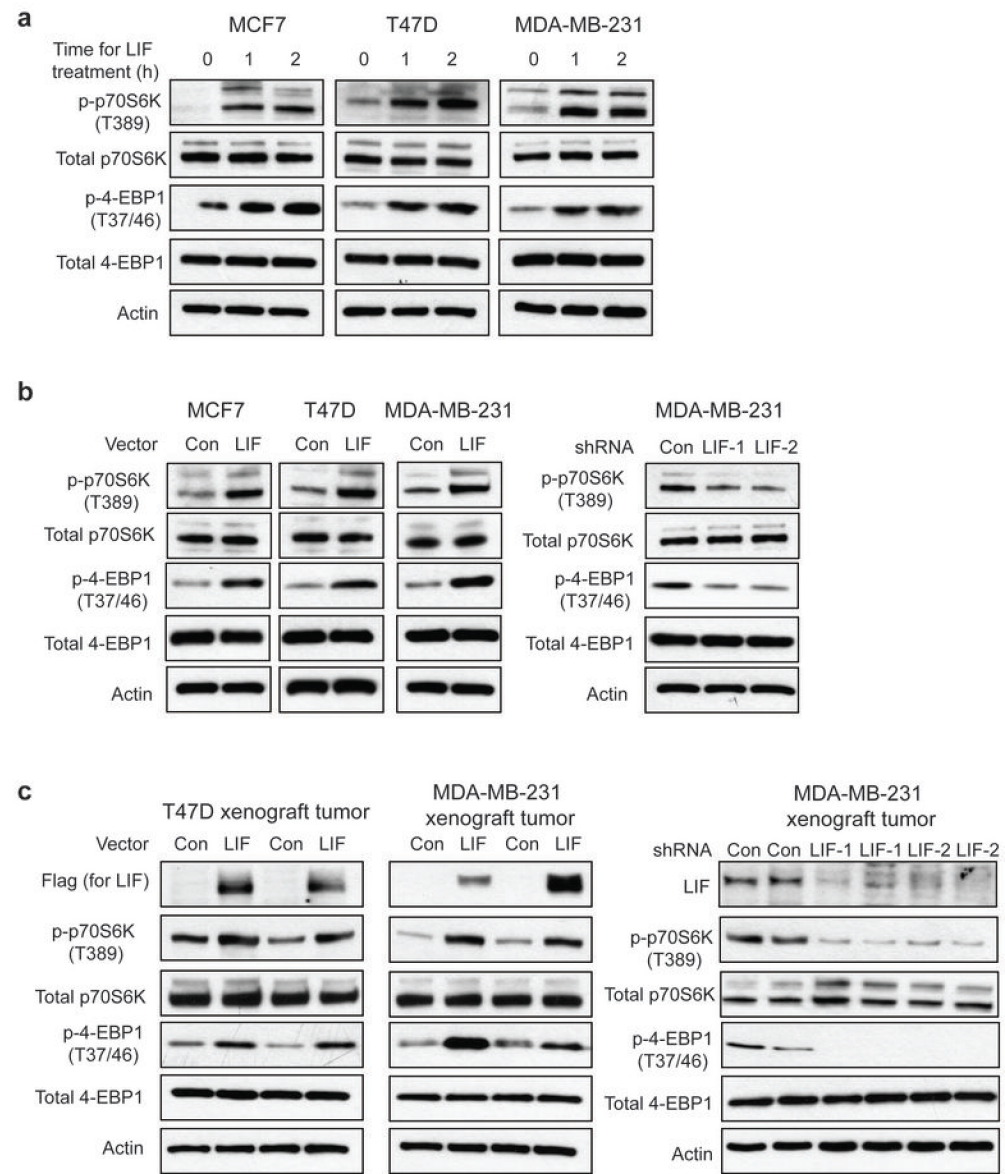
LIF activates the mTOR pathway in breast cancer cells (a) Exogenous LIF (100 ng/ml) increased the levels of phosphorylated p70S6K at Thr-389 (p-p70S6K) and phosphorylated 4EBP1 at Thr 37/46 (p-4EBP1) in MCF7, T47D and MDA-MB-231 cells. (b) Ectopic LIF expression in MCF7, T47D and MDA-MB-231 cells increased p-p70S6K and p-4EBP1 levels (left panels), whereas knockdown of LIF in MDA-MB-231 cells decreased p-p70S6K and p-4EBP1 levels (right panels). (c) Ectopic LIF expression increased p-p70S6K and p-4EBP1 levels in T47D and MDA-MB-231 xenograft tumors. Knockdown of LIF reduced p-p70S6K and p-4EBP1 levels in MDA-MB-231 xenograft tumors. The expression levels of LIF in xenograft tumors were confirmed by Western-blot assays.

The mTOR pathway is frequently activated in breast cancers. The activation of mTOR and the subsequent phosphorylation and activation of its downstream targets p70S6K and eIF4E binding protein 1 (4EBP1) play an important role in promoting cell growth, proliferation and metastasis in breast cancers [23-26]. We found that LIF activates the mTOR pathway in breast cancer cells. Exogenous LIF treatment increased the phosphorylation levels of p70S6K at Thr-389 (p-p70S6K) and 4EBP1 at Thr-37/46 (p-4EBP1), which represent the activity of p70S6K and 4EBP1, respectively, in T47D, MCF-7 and MDA-MB-231 cells (Fig. 3a). Similarly, ectopic LIF expression in these breast cancer cell lines increased p-p70S6K and p-4EBP1 levels (Fig. 3b). Furthermore, knock-down of LIF in MDA-MB-231 cells decreased p-p70S6K and p-4EBP1 levels (Fig. 3b). Consistently, T47D-LIF and MDA-MB-231-LIF xenograft tumors displayed much higher levels of p-p70S6K and p-4EBP1 than T47D-Con and MDA-MB-231-Con tumors (Fig. 3c). MDA-MB-231-LIFshRNA xenograft tumors displayed much lower levels of p-p70S6K and p-4EBP1 than MDA-MB-231-ConshRNA tumors (Fig. 3c).

**Figure 4:**
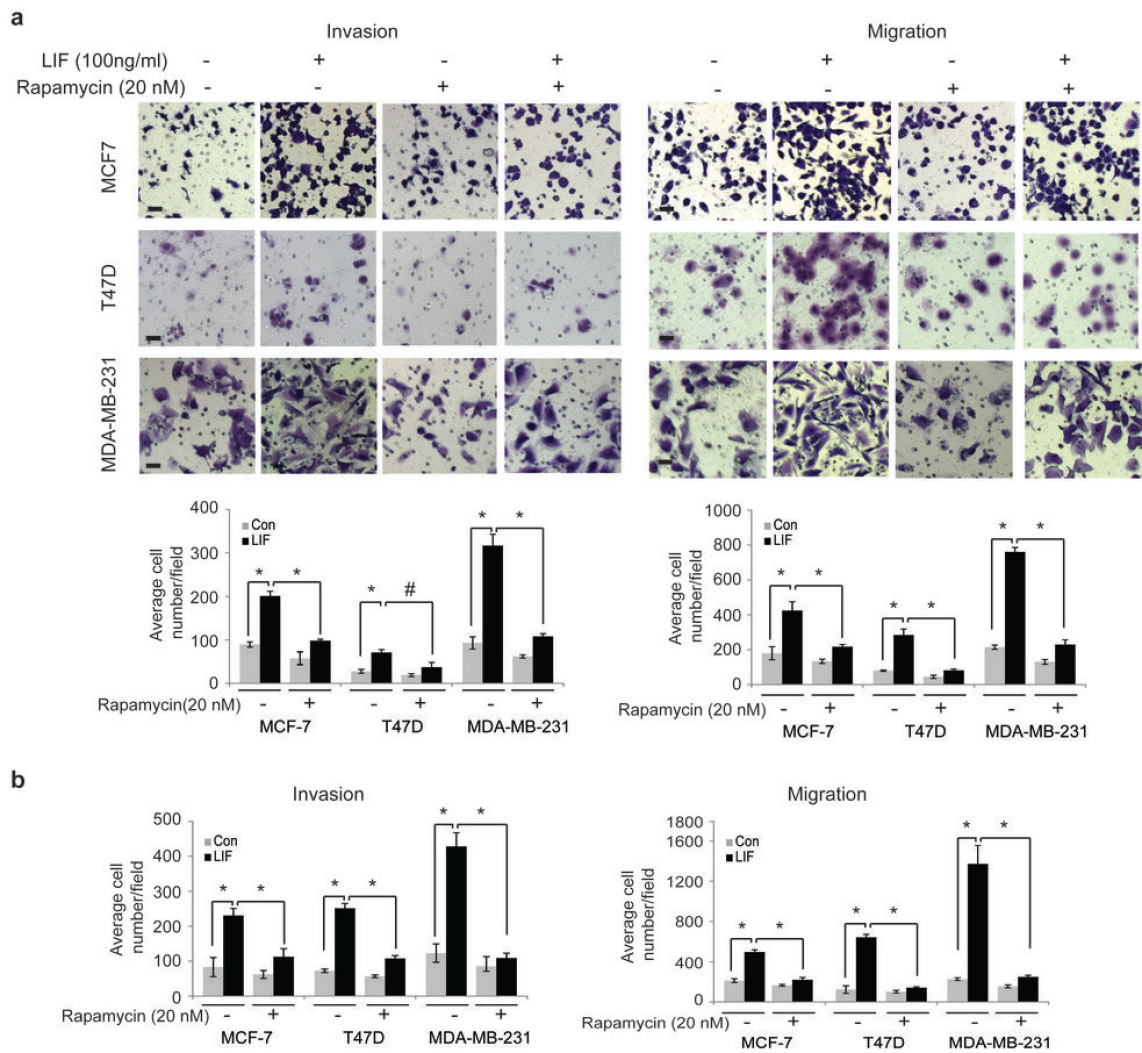
Blocking the mTOR signaling largely abolishes the promoting effect of LIF on invasion and migration of breast cancer cells (a) MCF7, T47D and MDA-MB-231 cells were treated with LIF (100 ng/ml) alone or in combination with rapamycin (20 nM). The invasion and migration abilities of cells were determined by trans-well assays. Upper panels: representative images; lower panels: quantifications of average number of cells/field. (b) MCF7-LIF, T47D-LIF, MDA-MB-231-LIF and their control cells were treated with or without rapamycin (20 nM). The invasion and migration abilities of cells were determined. Data are presented as mean ± SD (n=3). *: *p*<0.001; #: *p*<0.01 .

To investigate whether the activation of mTOR pathway by LIF contributes to the role of LIF in breast cancer metastasis, rapamycin, a highly specific mTOR inhibitor, was employed to block the mTOR pathway, and the effect of LIF on invasion and migration was determined. Rapamycin treatment largely blocked the promoting effect of both exogenous LIF and ectopically expressed LIF in cells on invasion and migration in MCF-7, T47D and MDA-MB-231 cells (Fig. 4a & b). Taken together, these results demonstrate that LIF activates the mTOR pathway, which contributes to the promoting effect of LIF on breast cancer metastasis.

### LIF activates the mTOR pathway through AKT in breast cancer cells

**Figure 5:**
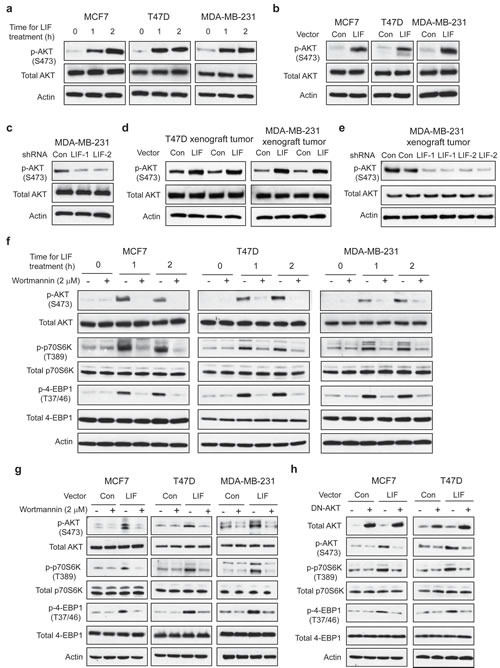
LIF activates the mTOR pathway through AKT in breast cancer cells (a) Exogenous LIF treatment (100 ng/ml) activated AKT activity in MCF7, T47D and MDA-MB-231 cells. The levels of total and phosphorylated AKT at Ser 473 (p-AKT-S473) were determined by Western-blot assays. (b) Ectopic LIF expression increased p-AKT-S473 levels in breast cancer cell lines. (c) Knockdown of LIF decreased p-AKT-S473 levels in MDA-MB-231 cells. (d) Ectopic LIF expression increased p-AKT-S473 levels in T47D and MDA-MB-231 xenograft tumors. (e) Knockdown of LIF reduced p-AKT-S473 levels in MDA-MB-231 xenograft tumors. (f&g) Blocking AKT activity by Wortmannin, an AKT inhibitor, largely abolished the effect of LIF on the mTOR signaling. (f) MCF7, T47D and MDA-MB-231 cells were treated with LIF (100 ng/ml) alone or in combination with wortmannin (2 μM) for the indicated time periods. (g) MCF7-LIF, T47D-LIF, MDA-MB-231-LIF and their control cells were treated with wortmannin (2 μM) for 24 h. (h) Blocking AKT activity by expressing a dominant negative AKT (DN-AKT) largely abolished the effect of LIF on the mTOR signaling. MCF7-LIF, T47D-LIF and their control cells were transduced with a dominant negative AKT (pLHCX-DN-AKT, K179M) expression vector.

It has been reported that LIF activates the AKT pathway in several different cell types, including human embryonic kidney 293T, liver Hep3B, and oligodendrocytes [7, 27]. We found that LIF activates the AKT pathway in breast cancer cells. As shown in Fig. 5a & b, both exogenous LIF treatment and ectopically expressed LIF in cells increased the phosphorylation of AKT at Ser-473 (p-AKT), which represents the activation of AKT, in MCF7, T47D and MDA-MB-231 cells. Knock-down of LIF decreased p-AKT in MDA-MB-231 cells (Fig. 5c). The activation of the AKT pathway by LIF was also observed in xenograft breast tumors. The levels of p-AKT were much higher in T47D-LIF and MDA-MB-231-LIF xenograft tumors than T47D-Con and MDA-MB-231-Con tumors, respectively (Fig. 5d). The levels of p-AKT were much lower in MDA-MB-231 LIFshRNA xenograft tumors than MDA-MB-231-con tumors (Fig. 5e).

The AKT pathway crosstalks with and activates the mTOR pathway [28], which raises the possibility that LIF activates the mTOR pathway through AKT. As shown in Fig. 5f & g, blocking AKT activity by wortmannin, a specific AKT inhibitor, largely abolished the activation of the mTOR pathway by LIF; the elevation of p-p70S6K and p-4EBP1 levels by both exogenous LIF treatment and ectopically expressed LIF was largely diminished in cells treated with wortmannin. Similarly, blocking the AKT activity by expressing a dominant negative AKT (DN-AKT) reduced the levels of p-p70S6K and p-4EBP1 in MCF7-LIF and T47D-LIF cells but not in MCF7-Con and T47D-Con cells (Fig. 5h). Consistently, while stable LIF expression in xenograft T47D tumors increased the levels of p-p70S6K and p-4EBP1, this effect of LIF was largely blocked in T47D tumors with co-expression of LIF and DN-AKT (Supplementary Fig. 2). LIF can activate multiple signaling pathways, including STAT-3 pathway, to mediate some of LIF's functions. Here, we also tested whether LIF activates the mTOR pathway through the STAT-3 pathway. As shown in Supplementary Fig. 3, exogenous LIF treatment dramatically increased the phosphorylation of STAT-3 at Thy705, which represents the activation of the STAT-3 pathway. However, blocking STAT-3 activity by employing Stattic, a specific STAT-3 inhibitor, did not have a significant effect on the activation of the mTOR pathway by LIF, suggesting that STAT-3 does not play a major role in the activation of the mTOR pathway by LIF. Taken together, our results demonstrate that LIF activates the mTOR pathway mainly through AKT.

### Blocking the AKT pathway largely abolishes the promoting effect of LIF on tumorigenesis and metastasis of breast cancer

**Figure 6:**
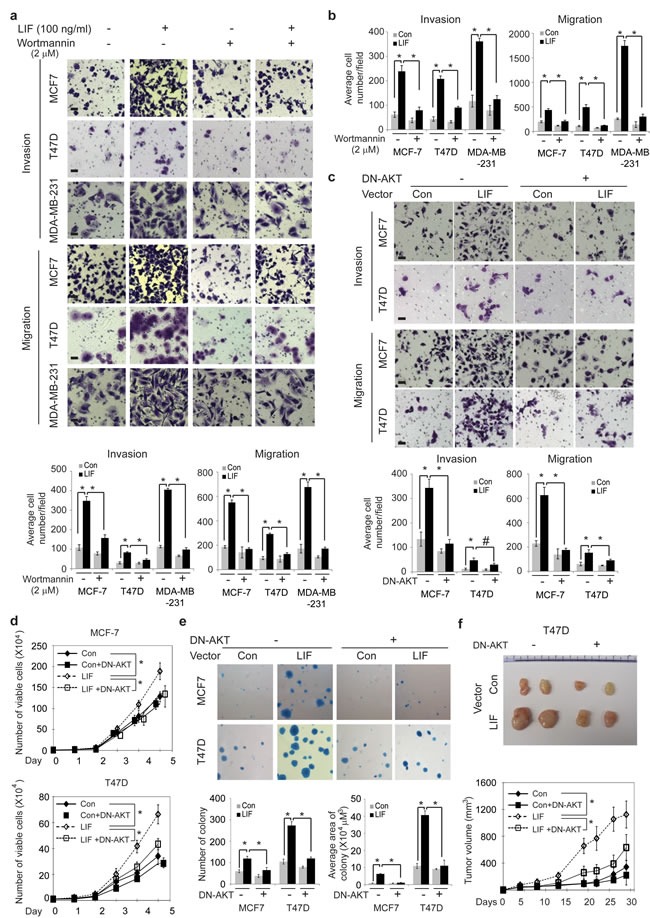
Blocking the AKT signaling inhibits the promoting effect of LIF on tumorigenesis and metastasis in breast cancer cells (a) MCF7, T47D and MDA-MB-231 cells were treated with LIF (100 ng/ml) alone or in combination with wortmannin (2 μM), and the invasion and migration abilities of cells were determined. (b) MCF7-LIF, T47D-LIF, MDA-MB-231-LIF and their control cells were treated with or without wortmannin (2 μM), and the invasion and migration abilities of cells were determined. (c) Blocking AKT activity by the expression of DN-AKT largely abolished the effect of LIF on invasion and migration abilities of MCF7-LIF and T47D-LIF cells. (d) The expression of DN-AKT largely abolished the promoting effect of LIF on proliferation in MCF7-LIF and T47D-LIF cells. (e) The expression of DN-AKT largely blocked the promoting effect of LIF on anchorage-independent growth in soft agar in MCF7-LIF and T47D-LIF cells. (f) The expression of DN-AKT largely blocked the promoting effect of LIF on growth rate of xenograft T47D-LIF tumors. Data are presented as mean ± SD. n=3 for a–e; n=10 for f. Scale bar: 50 μM. *: *p*<0.001; #: *p*<0.01.

To further determine whether the activation of AKT-mTOR pathway contributes to the effect of LIF on breast cancer tumorigenesis and metastasis, above-mentioned breast cancer cell lines were treated with LIF along with or without wortmannin to block AKT pathway. As shown in Fig. 6a & b, wortmannin treatment largely blocked the promoting effect of both exogenous LIF and ectopically expressed LIF on invasion and migration in MCF-7, T47D and MDA-MB-231 cells. Similarly, expressing DN-AKT in MCF7 and T47D cells to block AKT activity largely blocked the promoting effect of ectopically expressed LIF on invasion and migration in cells (Fig. 6c). Furthermore, expression of DN-AKT greatly reduced the promoting effect of LIF on cell proliferation and anchorage-independent cell growth in soft agar in MCF7 and T47D cells (Fig. 6d & e). Expression of DN-AKT also largely abolished the promoting effect of LIF on the growth of xenograft tumors formed by T47D cells (Fig. 6f). Taken together, these results demonstrate that LIF activates the AKT-mTOR pathway, which in turn promotes tumorigenesis and metastasis of breast cancer.

### LIF correlates with poor prognosis in breast cancer patients

**Figure 7:**
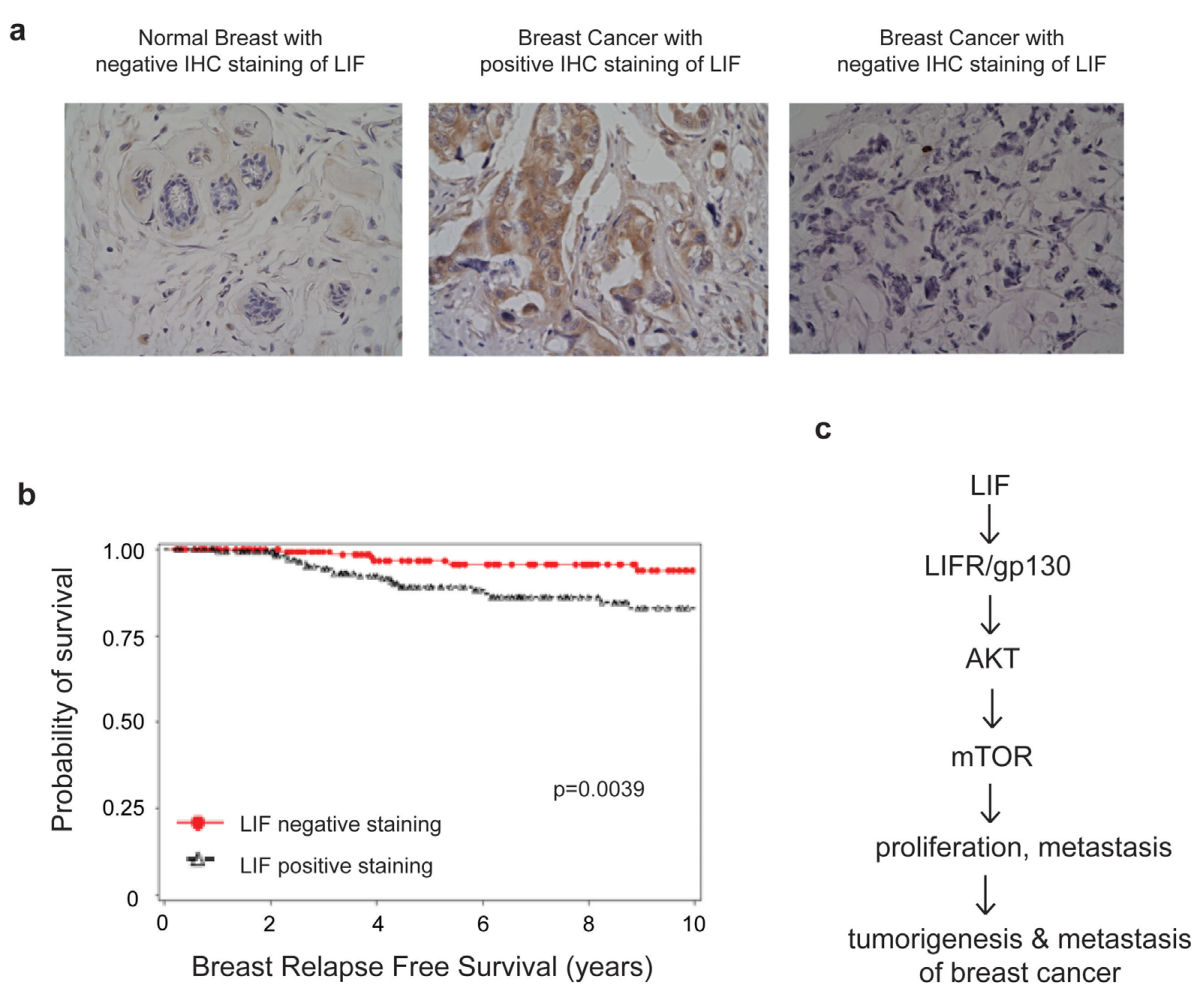
High LIF expression levels are associated with a poor relapse free survival of breast cancer patients (a) The expression of LIF in a cohort of 374 human breast cancer specimens was determined by IHC staining of LIF. Representative images of LIF IHC staining in normal and breast cancer specimens are shown. (b) Kaplan-Meier curves for relapse free survival in breast cancer patients with or without positive LIF staining. Positive LIF staining (>10% cells stained with LIF) in breast cancer specimens is significantly correlated with a poorer relapse free survival and a higher breast cancer relapse risk (*p*=0.0039). (c) Schematic model depicting the activation of the AKT-mTOR signaling by LIF which in turn promotes proliferation and metastasis of breast cancer.

To evaluate the clinical importance of LIF in breast cancer, a cohort of 374 breast cancer patients was employed. The expression levels of LIF were determined in the tissue microarray containing the breast tumor tissues from these patients by using IHC staining. 54.8% tumor samples were positive for LIF staining. Representative LIF positive and negative staining images were shown in Fig. 7a. The relationship between LIF expression levels and clinicopathological variables of breast cancer was analyzed. As summarized in Table 1, the expression levels of LIF were associated with age at diagnosis (p=0.001), estrogen receptor (ER, p=0.025) and progesterone receptor (PR, p=0.002). Notably, employing Kaplan-Meier survival analysis, we found that LIF expression levels were correlated with relapse free survival of breast cancer patients (p=0.0039). The prognosis analysis demonstrates that higher expression of LIF had a poorer relapse free survival in breast cancer patients (Fig. 7b), suggesting that LIF could be a prognostic marker for poor prognosis of breast cancer patients.

**Table 1:** The relationship between LIF expression and clinicopathological variables of breast cancer

Clinicopathological variables	LIF Expression		P
	Negative (Number)	Positive (Number)	
Age			0.001
≤40	16	45	
>40	153	160	
Race			0.094
White	147	167	
Black	16	34	
Others	6	4	
Histology			0.159
Ductal	142	182	
Lobular	17	10	
Others	10	12	
Tumor size			0.179
≤2cm	123	137	
>2cm	31	49	
Nodal status			0.757
Negative	72	95	
Positive	28	33	
Unknown	69	77	
ER			0.025
Negative	81	74	
Positive	87	128	
PR			0.002
Negative	92	77	
Positive	77	125	
HER2			0.307
Negative	141	162	
Positive	27	41	

## DISCUSSION

LIF is a cytokine with a wide array of functions. LIF plays an essential role in embryonic implantation and maintaining pluripotentiality of murine stem cells. Recently, we identified that LIF is a novel p53 target gene, and importantly, LIF mediates p53's role in embryonic implantation [29-31]. LIF is also involved in bone formation, neuronal survival, and acute immune response to inflammation [32, 33]. The role of LIF in cancer and its underlying mechanisms are not well-understood with limited studies suggesting that LIF may play a role in tumorigenesis.

Results from this study demonstrate that LIF promotes the growth and metastasis of breast cancer. LIF promotes proliferation, anchorage-independent growth in soft agar of breast cancer cells, and the growth rate of xenograft breast tumors. LIF also promotes metastasis of breast cancer cells as determined by *in vitro* trans-well and *in vivo* lung and distant metastatic assays. Both exogenous LIF and LIF expressed in cells show promoting effects on tumorigenesis and metastasis of breast cancer, suggesting that LIF functions in both autocrine and paracrine manners. Furthermore, the promoting effect of LIF on tumorigenesis and metastasis of breast cancer is independent of ER status; similar effects of LIF were observed in both ER-positive breast cancer cells (MCF7 and T47D) and ER-negative breast cancer cells (MDA-MB-231).

LIF binds to its receptor complex composed of LIFR and gp130 to activate the LIF signaling pathway. It has been reported that LIF selectively activates JAK/STAT3, PI3K/AKT, MAPK, and/or ERK1/2 pathways in different tissues, cell types or conditions. Results in this study demonstrate that LIF activates the mTOR pathway in breast cancer. The mTOR pathway is frequently activated in various cancers, including breast cancer, which plays a critical role in tumor growth and metastasis [34-36]. Importantly, blocking mTOR activity largely abolished the promoting effect of LIF on breast cancer metastasis, suggesting that the activation of mTOR pathway mediates the promoting effect of LIF on breast cancer tumorigenesis and metastasis. Further investigation on the mechanism by which the mTOR pathway is activated by LIF shows that the activation of mTOR is largely mediated by AKT. Blocking AKT activity by wortmannin or the expression of DN-AKT largely abolished the activation of mTOR by LIF, and more importantly, largely abolished the promoting effect of LIF on tumorigenesis and metastasis of breast cancer. These findings demonstrate that LIF promotes tumorigenesis and metastasis of breast cancer through the AKT-mTOR signaling pathway (Fig. 7c).

It is currently unclear whether LIF activates the AKT-mTOR pathway to promote tumorigenesis and metastasis in other types of cancers. During the preparation of this paper, a very recent study reported that exogenous LIF activates the mTOR pathway in nasopharyngeal carcinoma cell lines, which is consistent with our findings [37]. It will be interesting to investigate whether LIF also activates the mTOR pathway through AKT in nasopharyngeal cancer, whether the activation of LIF-AKT-mTOR pathway exists in other types of tumors, and plays an important role in tumorigenesis in future studies.

To date, the association between LIF and prognosis of breast cancer has not been reported. Our results show that higher LIF expression is associated with poorer relapse free survival of breast cancer patients, indicating that LIF could be an important prognostic marker for breast cancer patients. LIF functions as a cytokine protein. Therefore, using antibody against LIF to block LIF function or blocking LIF receptor complex are potential strategies for breast cancer therapy. Furthermore, given the effect of LIF expression on the AKT-mTOR signaling, this pathway may serve as a novel therapeutic target to improve outcomes in breast tumors with LIF overexpression.

In summary, results from this study demonstrate that LIF plays a vital role in promoting growth and metastasis of breast cancer. This function of LIF in breast cancer is mainly mediated by the AKT-mTOR pathway. Results from this study have the direct potential to develop LIF as an important biomarker for prognosis of breast cancer and a therapeutic target for breast cancer, especially for those with LIF overexpression.

## MATERIAL AND METHODS

### Cell culture and cell treatments

Human breast cancer cell lines MCF7, T47D, MDA-MB-231, HS578T, MDA-MB-468, SK-Br-3, BT474, and NIH3T3 fibroblasts were obtained from ATCC. Cells with stable LIF overexpression were established by transduction of a retroviral LIF expression vector (pLPCX-LIF) and selected by puromycin. To knockdown endogenous LIF expression in cells, two validated shRNA vectors against LIF (SHCLNDNM_002309, Sigma) were transduced into cells and selected by puromycin. Recombinant human LIF protein was purchased from Millipore. Cells were treated with Rapamycin (Cell Signaling Technology), Wortmannin (Cell Signaling Technology) and Stattic (Sigma) for various time periods before being harvested for further analysis.

### Plasmids construction

The pLPCX-LIF vector expressing LIF with C-terminal flag was constructed by amplifying LIF cDNA using following primers:5'AAGCTTATGAAGGTCTTGGCGGCAGGAG-3'; 5'-TGAATTCGCGAAGGCCTGGGCCAA-3'. LIF fragment was inserted into p3XFlag-CMV-14 vectors, then subcloned into pLPCX vectors along with the flag tag. The pLHCX-DN-AKT vector expressing a dominant negative AKT (DN-AKT; K179M) was constructed by subcloning the DN-AKT fragment from pLNCX-AKT1 K179M (Addgene) into the pLHCX vectors [38].

### Taqman real-time PCR

Total RNA was prepared by using an RNeasy kit (Qiagen). All primers were purchased from Applied Biosystems. Real-time PCR was done in triplicate with TaqMan PCR mixture (Applied Biosystems).

### Western-blot assays

Standard Western-blot assays were performed as previsouly described [39]. Anti-human LIF antibody (AF250-NA) was purchased from R&D. Antibodies against phospho-p70S6 kinase (p-p70S6K, Thr-389), total p70S6K, phospho-AKT (Ser-473), phospho-4EBP1 (Thr-37/46), total 4EBP1 and phospho-STAT3 (Thy-705) were purchased from Cell Signaling Technology. Antibodies against LIFR (sc-659), total AKT (sc-1618) and total STAT3 (sc-482) were purchased from Santa Cruz Biotechnology. Anti-gp130 antibody was purchased from Millipore. Anti-actin and anti-FLAG antibodies were purchased from Sigma.

### Cell migration and invasion assays

The trans-well system (24 wells, 8 μM pore size, BD Biosciences) was employed for cell migration and invasion assays as previously described [40]. In brief, cells in serum-free medium were seeded into upper chambers coated with or without matrigel (BD Biosciences) for invasion and migration assays, respectively. The lower chamber was filled with 1:1 mix of medium supplemented with 10% FBS and NIH 3T3 cell-conditioned medium. Cells on the lower surface were fixed, stained and counted after culturing 24 h for MCF7 and MDA-MB-231 cells, and 48 h for T47D cells. 8×10^4^ MCF7 cells, 1.5×10^5^ T47D cells, and 5×10^4^ MDA-MB-231 cells were used for invasion assays; 5×10^4^ MCF7 cells, 1×10^5^ T47D cells, and 3×10^4^ MDA-MB-231 cells were used for migration assays.

### Anchorage-independent growth assays

Anchorage-independent growth assays were performed as previously described [41]. In brief, cells were seeded in 6-well culture plates coated with media containing 0.6% agarose, and cultured in media containing 0.3% agarose. Colonies were stained and counted after 2-3 weeks.

### Xenograft tumorigenicity assays

Cells (5 × 10^6^ in 0.2 mL PBS) were injected subcutaneously (s.c.) into 6-week-old BALB/c female athymic nude mice (Taconic). For xenograft tumorigencity assays of T47D and MCF-7 cells, a 17β-estradiol pellet (Innovative Research of America) was implanted into each mouse 2 days before cell injection. After injection, mice were examined and tumor volumes were measured 3 times/week for 2-4 weeks. Tumor volume = 1/2 (length × width^2^). Tumor samples were processed for routine histopathological examination.

### *In vivo* metastasis assays

Cells (2 × 10^6^ in 0.1mL PBS) were injected into BALB/c female nude mice via tail vein. The mice were sacrificed at indicated time after the inoculation. The numbers of lung and distant metastatic tumors were counted under a dissecting microscope and confirmed by histopathological analysis. Mouse experiments were approved by the University Institutional Animal Care and Use Committee.

### Tissue microarray and clinical information of breast cancer patients

Tissue microarray comprising duplicate cores of tumors from 374 breast cancer patients was used for this study. The de-identified clinical information of these patients was obtained from the patient database that has been previously described [42, 43]. In brief, none of the patients in this study received chemotherapy or irradiation therapy prior to the surgery. They were treated with breast conserving surgery with or without axillary lymph node dissection. Following surgery, adjuvant systemic therapy was administered as clinically indicated in accordance with standard clinical practice. The median follow up time is 7.9 years. This study was reviewed and approved by University Human Investigations Committee Institutional Review Board.

### Immunohistochemistry (IHC) assays for LIF

IHC staining of LIF was performed as previously described [42-44]. In brief, tissue sections were deparaffinized and antigen retrieval was achieved by Target Retrieval Solution (DAKO). Tissue sections were incubated with anti-LIF antibody (MAB250, R&D, 1:20 dilution) overnight at 4°C, followed with a biotinylated secondary antibody staining. Immunoreactivity was detected by using a Vectastain Elite ABC kit (Vector). Known positive controls were included in each experiment, and negative controls were obtained by omitting the primary antibody. The tumor was considered positive for LIF staining when more than 10% of tumor cells were positive for LIF staining.

### Statistical analysis

A computer program package SAS (Version 9.1, SAS Institute) was employed to manage the patient database and analyze the statistic difference. The relationship between LIF and clinicopathological variables was analyzed by standard Chi-square test. The Kaplan–Meier analysis was used to assess the relationship between LIF and the survival of patients. The differences were assessed by the log-rank test. The statistical differences in xenograft tumor growth among groups were analyzed by ANOVA, followed by Student's t-test. All other *p* values were obtained using Student's t-test. *p*<0.05 was considered statistically significant.

## SUPPLEMENTARY FIGURES


