# Supplementary material for: LIF promotes tumorigenesis and metastasis of breast cancer through the AKT-mTOR pathway
Source: Oncotarget. 2014 Feb 11;5(3):788–801. doi: 10.18632/oncotarget.1772 (PMC3996668; doi:10.18632/oncotarget.1772)

Supplementary materials

For

LIF promotes tumorigenesis and metastasis of breast cancer through the AKT-mTOR pathway

Figure legends

Supplementary Figure 1. The expression levels of LIFR and gp130 in a panel of breast cancer cells. The mRNA expression levels of LIFR (**a**) and gp130 (**b**) were examined in a panel of breast cancer cells by using Taqman real-time PCR. The expression levels of LIFR and gp130 were normalized with actin. Data are presented as mean ± SD (n=3).

Supplementary Figure 2. Blocking AKT activity by the expression of DN-AKT largely abolishes the effect of LIF on the mTOR pathway *in vivo*. The levels of total and phosphorylated AKT at Ser-473 (p-AKT), total and phosphorylated p70S6K at Thr-389 (p-p70S6K), total and phosphorylated 4EBP1 at Thr 37/46 (p-4EBP1) were determined in xenograft tumors formed by T47D cells with or without ectopic LIF expression and/or DN-AKT by Western-blot assays.

Supplementary Figure 3. The activation of the mTOR pathway by LIF is independent of the STAT-3 signaling in breast cancer cells. Exogenous LIF (100 ng/ml) activated the STAT-3 and mTOR pathways. Blocking STAT-3 activity had no significant effect on the activation of mTOR by LIF. Cells were treated with LIF along with or without static (3 μM), a specific STAT-3 inhibitor, for the indicated

time periods. The levels of total and phosphorylated STAT-3 at Thy-705 (p-STAT-3), total and phosphorylated p70S6K at Thr-389 (p-p70S6K), total and phosphorylated 4EBP1 at Thr 37/46 (p-4EBP1) were determined by Western-blot assays.

Supplementary Fig. 1

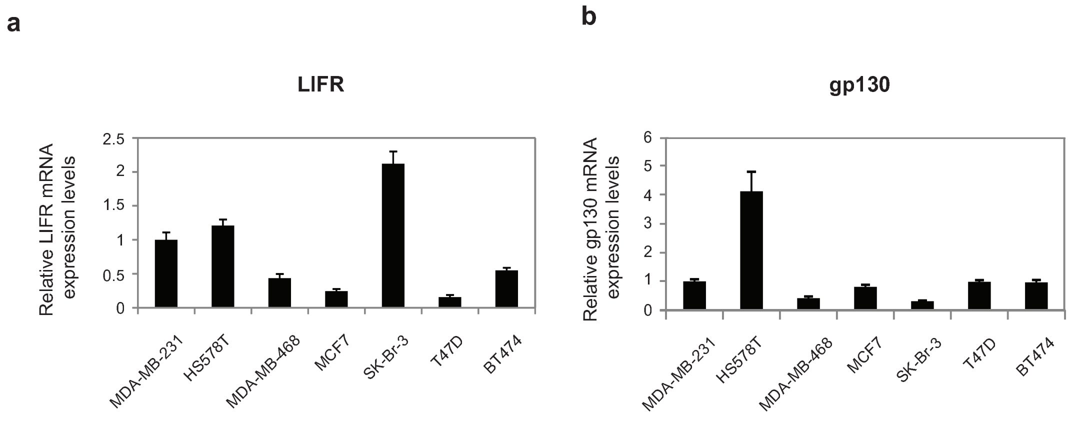

Supplementary Fig. 2

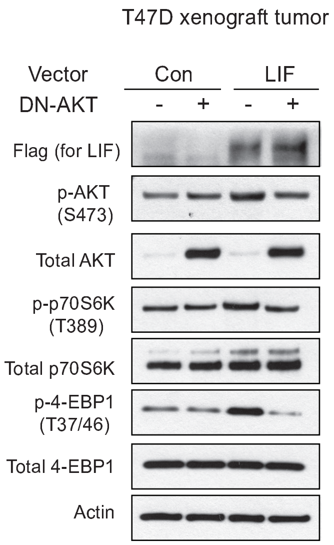

Supplementary Fig. 3

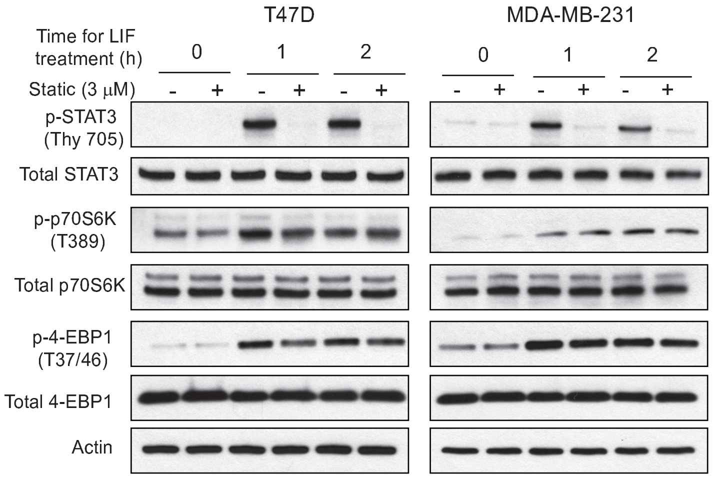

Supplement: Supplementary file 1 [file oncotarget-05-788-s001.pdf]
